# Cost-Effectiveness of [^99m^Tc]Tilmanocept Relative to [^99m^Tc]Sulfur Colloid for Sentinel Lymph Node Biopsy in Early Stage Oral Cavity Cancer

**DOI:** 10.1245/s10434-023-13937-y

**Published:** 2023-08-09

**Authors:** Karen Y. Choi, Qiang Hao, Kathryn Carlisle, Christopher S. Hollenbeak, Stephen Y. Lai

**Affiliations:** 1 Penn State College of Medicine, Hershey Medical Center, Hershey, PA USA; 2 Department of Otolaryngology Head and Neck Surgery, Penn State Hershey Medical Center, Hershey, PA USA; 3 Department of Health Policy and Administration, Penn State University, University Park, PA USA; 4 Department of Head and Neck Surgery, University of Texas MD Anderson Cancer Center, Houston, TX USA; 5 Department of Molecular and Cellular Oncology, University of Texas MD Anderson Cancer Center, Houston, TX USA; 6 Department of Radiation Oncology, University of Texas MD Anderson Cancer Center, Houston, TX USA

## Abstract

**Background:**

Several studies have demonstrated varying rates of efficacy, reliability, and sensitivity of sentinel lymph node biopsy (SLNB) in identifying occult nodal disease for early stage oral cavity squamous cell carcinoma (OCSCC) depending on the radionuclide agent utilized. No head-to-head comparison of cost or clinical outcomes of SLNB when utilizing [^99m^Tc]tilmanocept versus [^99m^Tc]sulfur colloid has been performed. The goal of this study was to develop a decision model to compare the cost-effectiveness of [^99m^Tc]tilmanocept versus [^99m^Tc]sulfur colloid in early stage OCSCC.

**Patients and Methods:**

A decision model of disease and treatment as a function of SLNB was created. Patients with a negative SLNB entered a Markov model of the natural history of OCSCC parameterized with published data to simulate five states of health and iterated over a 30-year time horizon. Treatment costs and quality-adjusted life-years (QALYs) for each health state were included. The incremental cost-effectiveness ratio (ICER) was then estimated using $100,000 per additional QALY as the threshold for determining cost-effectiveness.

**Results:**

The base case cost-effectiveness analysis suggested [^99m^Tc]tilmanocept was more effective than [^99m^Tc]sulfur colloid by 0.12 QALYs (7.06 versus 6.94 QALYs). [^99m^Tc]Tilmanocept was more costly, with a lifetime cost of $84,961 in comparison with $84,264 for sulfur colloid, however, the overall base case ICER was $5859 per additional QALY, well under the threshold for cost-effectiveness. Multiple one-way sensitivity analyses were performed, and demonstrated the model was robust to alternative parameter values.

**Conclusion:**

Our analysis showed that while [^99m^Tc]tilmanocept is more costly upfront, these costs are worth the additional QALYs gained by the use of [^99m^Tc]tilmanocept.

Historically, the clinically N0 neck in early stage oral cavity squamous cell carcinoma (OCSCC) has been managed with elective neck dissection (END) for tumors with depth greater than 4 mm given the 20–30% risk of occult nodal metastasis.^1,2^ In a landmark prospective randomized clinical trial, the survival benefit of elective neck dissection over watchful waiting was confirmed, demonstrating improved disease-free and overall survival.^3^ More recently, there has been much debate over the optimal management of the clinically N0 neck given that 70–80% of patients may be unnecessarily treated with an elective neck dissection. To address this clinical dilemma, the introduction of sentinel lymph node biopsy (SLNB) as a staging method for the clinically N0 neck has been monumental in potentially changing the landscape of how early stage OCSCC are managed. While SLNB has been well established as standard of care for management of cutaneous malignant melanoma and breast cancer, this was only recently adopted into OCSCC management in the past two decades.^4,5^ Numerous studies have validated the use of SLNB for early stage oral cavity cancer, with one meta-analysis of 26 studies noting an overall pooled negative predictive value of SLNB for oral cavity tumors of 96% (95% CI 94–99%) and a sensitivity of 94% (95% CI 89–98%).^6^ A more recent meta-analysis that included 66 studies reported a similar pooled negative predictive value of 94% (95% CI 93–95%), but a slightly lower pooled sensitivity of 87% (95% CI 85–89%) than the literature.^7^ SLNB has been shown to decrease morbidity, surgical time, and length of hospital stay compared with END.^8,9^ Furthermore, numerous investigations have demonstrated improved cost savings when utilizing SLNB over END for early stage OCSCC.^10–12^

Several radionuclide agents are utilized for SLNB, and in the USA, the most common agents are [^99m^Tc]sulfur colloid and [^99m^Tc]tilmanocept. Despite excellent negative predictive values as noted earlier, false negative rates are more variable depending on the oral cavity subsite and agent used, approaching close to 10%.^13^ Sulfur colloid is a larger particle than tilmanocept, measuring ~ 200 nm and travels via passive diffusion through the lymphatic network. [^99m^Tc]Tilmanocept is a novel agent that was given an FDA indication in 2014 for use in oral cavity and is much smaller, with a diameter of 7 nm. It contains a mannose moiety that allows for specific targeting of the CD206 mannose-binding receptors expressed specifically on the surface of reticulendothelial cells within lymph nodes.^14^ The primary advantages of this approach include improved primary injection site clearance due to the smaller size and increased uptake and retention in the sentinel lymph nodes due to specific receptor targeting. In a prospective multi-institutional study assessing the accuracy of [^99m^Tc]tilmanocept in SLNB for OCSCC, the false negative rate was reported to be 2.56% (95% CI 0.06–13.49),^15^ in comparison with the previously reported false negative rate of 9.8% (95% CI 2.7–23.1) with [^99m^Tc]sulfur colloid.^13^ Furthermore, the false negative rate was favorable for notoriously difficult subsites such as the floor of mouth, with a rate of 0% when using [^99m^Tc]tilmanocept compared with a rate of 25% with [^99m^Tc]sulfur colloid.^13,15^ Despite the apparent advantages of [^99m^Tc]tilmanocept, it is significantly more costly, with average cost of the agent ranging close to $700 compared with average cost of around $100 for [^99m^Tc]sulfur colloid. As a result, many hospitals are wary to incur the upfront cost of [^99m^Tc]tilmanocept given the overall high negative predictive value afforded by [^99m^Tc]sulfur colloid. The goal of this study was to create a decision model that allowed a direct comparison of the cost-effectiveness of [^99m^Tc]tilmanocept to [^99m^Tc]sulfur colloid when utilized for lymphoscintigraphy in early stage OCSCC.

## Patients and Methods

**Fig. 1:**
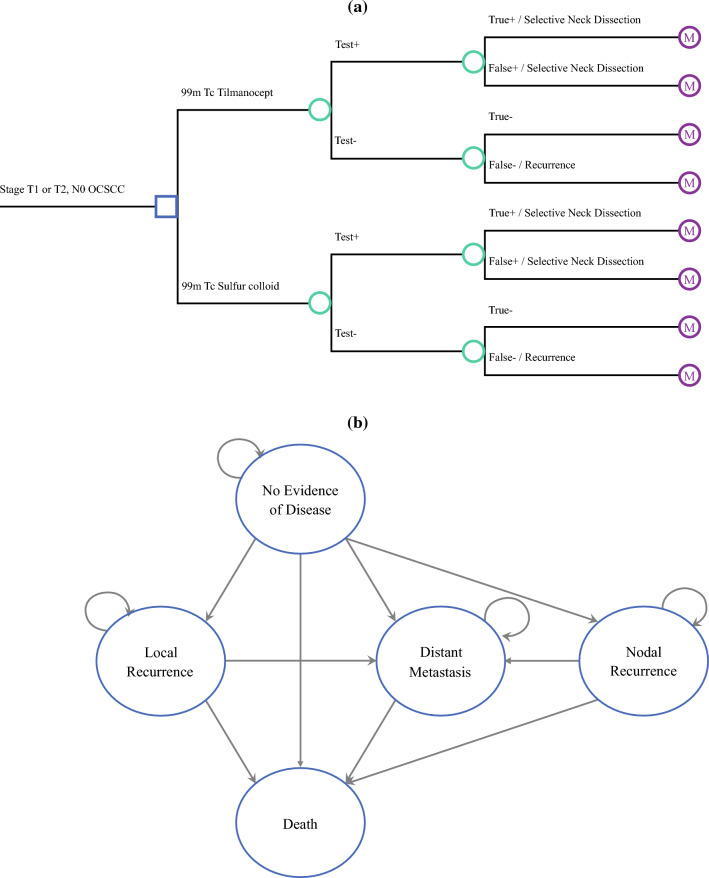
**a** Decision tree model for choice of agent for sentinel lymph node biopsy, model of disease and treatment designed as a function of sentinel lymph node biopsy; **b** Markov model of natural history of OCSCC, model parameterized from relevant literature to simulate five states of health in OCSCC and iterated in 1-year cycles over a 30-year time horizon; *OCSCC* oral cavity squamous cell carcinoma

To compare the cost-effectiveness of [^99m^Tc]tilmanocept and [^99m^Tc]sulfur colloid use for sentinel lymph node biopsy (SLNB) in the management of patients with early stage (T1/T2) clinically node-negative (N0) oral cavity squamous cell carcinoma (OCSCC), we created a decision tree model of disease and treatment as a function of SLNB. Figure 1a presents the structure of the decision tree. At the decision node, the surgeon decides between SLNB using either [^99m^Tc]tilmanocept or [^99m^Tc]sulfur colloid. The primary tumor was assumed to be resected at the time of SLNB. The SLNB produces a positive or negative result. Patients with a positive SLN were assumed to undergo subsequent completion neck dissection.^3,16^ Patients with a negative SLN test result were modeled to undergo surveillance of the neck for further treatment.^3,16^ After this point, patients entered a Markov model of the natural history of OCSCC.

The Markov model was parameterized using data from relevant literature to simulate five health states in this patient population, including no evidence of disease (NED), local recurrence, nodal recurrence, distant metastasis, and death.^3,16,17^ The Markov model was iterated in 1-year cycles over a 30-year time horizon.^16^ Figure 1b presents how patients flow within the Markov model. Our model assumed that all patients received corresponding treatments based on their SLNB test results and entered the Markov model with no evidence of disease.^3,16,17^ Outcomes from D’Cruz et al. and Acevedo et al. were used to parameterize the transitions from NED to local and nodal recurrence, which involve treatment with salvage surgery and adjuvant therapy, as well as transitions to distant metastases, which involved palliative chemotherapy.^3,16^

### Model Parameters

Table 1 presents transition probabilities that were drawn from the most recent relevant studies.^3,15,16,18–29^ When multiple studies estimated transition probabilities, we computed an average across all studies. [^99m^Tc]Tilmanocept and [^99m^Tc]sulfur colloid were assumed to have the same specificity, but the literature suggests [^99m^Tc]tilmanocept has a higher sensitivity, which is the primary driver of differences in outcomes and cost-effectiveness between the two test strategies.^15,18–25^ Disease progression was largely modeled after Acevedo et al., with some information from other relevant studies.^3,15,16,18–29^ Transitions to recurrent states had the same probabilities except for patients with a false negative SLNB result, who had approximately four times higher probability of transitioning from NED to nodal recurrence and about twice the probability of transitioning from NED to dead. They also had lower probabilities of transitioning from NED to local recurrence and distant metastasis.^3,16^

**Table 1:** Decision model parameters, base case parameter values and plausible range used in sensitivity analyses

Parameter	Base case		Range for sensitivity analysis		Sources
			Low	High	
*Specificity*					
[^99m^Tc]Tilmanocept	0.990		0.700	1.000	^15,25^
[^99m^Tc]Sulfur colloid	0.990		0.700	1.000	^18–24^
*Sensitivity*					
[^99m^Tc]Tilmanocept	0.979		0.750	1.000	^15,25^
[^99m^Tc]Sulfur colloid	0.727		0.500	1.000	^18–24^
Prevalence of metastasis	0.280		0.200	0.400	^26–28^
*Disease progression*	TP, TN, FP	FN			
NED → NED	0.869	0.749	0.500	0.990	^3,16^
NED → LR	0.032	0.007	0.001	0.100	^3,16^
NED → NR	0.035	0.137	0.010	0.250	^3,16^
NED → DM	0.004	0.003	0.001	0.050	^3,16^
NED → Dead	0.061	0.104	0.010	0.250	^3,16^
LR → LR	0.614	0.614	0.500	0.750	^16,29^
LR → DM	0.286	0.286	0.100	0.500	^16,29^
LR → Dead	0.100	0.100	0.010	0.250	^16^
NR → NR	0.589	0.589	0.400	0.750	^16,29^
NR → DM	0.286	0.286	0.150	0.500	^16,29^
NR → Dead	0.125	0.125	0.050	0.250	Assumption
DM → DM	0.837	0.837	0.750	0.950	Assumption
DM → Dead	0.163	0.163	0.050	0.250	Assumption

*TP* true positive, *TN* true negative, *FP* false positive, *FN* false negative, *NED* no evidence of disease, *LR* local recurrence, *NR* nodal recurrence, *DM* distant metastasis

Table 2 presents treatment costs and QALY for health states in the Markov model. This study estimates costs from the perspective of a third-party payer. Costs of [^99m^Tc]tilmanocept and [^99m^Tc]sulfur colloid were estimated on the basis of reported hospital acquisition costs. Costs and QALYs for primary oral tumor resection, concurrent neck dissection at time of primary resection, stand-alone salvage neck dissection, and radiation therapy were estimated from primary literature, with costs adjusted for inflation to 2020 US dollars using the Consumer Price Index.^16,30–33^ The cost of chemotherapy with radiation therapy and for metastatic disease were determined from wholesale drug pricing coupled to chemotherapy administration costs from the Health Care Utilization Project.^16,34–36^ Among patients with a positive test result, 49% were assumed to receive radiation therapy and 15% were assumed to receive chemotherapy.^3,16^ In addition, patients who entered the health state “death” were charged end of life (EOL) care costs that were estimated from the literature.^16,37^ All costs and QALYs in this study were discounted at 3% per year.^16^

**Table 2:** Costs and utilities for health states in the Markov model, costs for agents estimated from reported hospital acquisition costs and all other costs estimated from third-party payer perspective

Parameter	Base case	Range for sensitivity analysis		Sources
		Low	High	
*Cost*				
[^99m^Tc]Tilmanocept	$628	$400	$1500	Internal
[^99m^Tc]Sulfur colloid	$105	$10	$200	Internal
Primary oral tumor resection	$28,512	$18,466	$40,992	^16,30^
Concurrent neck dissection at time of primary resection	$3946	$1326	$7970	^16,31^
Stand-alone salvage neck dissection	$29,366	$26,078	$32,973	^16,30^
Radiation therapy	$24,693	$16,099	$35,708	^16,32^
Chemotherapy (cisplatin with radiation therapy)	$7684	$4926	$11,018	^16,34,35^
Chemotherapy (for metastatic disease)	$36,619	$23,478	$52,476	^16,34,36^
Remission (NED)	$1579	$1017	$2257	^16,54^
Remission (others)	$1071	$690	$1531	^16,54^
EOL care	$11,101	$7154	$15,852	^16,37^
*Utility*				
Primary oral tumor resection	0.913	0.301	1	^16,17^
Neck dissection	− 0.072	− 0.038	− 0.116	^16,55^
Salvage treatment	− 0.238	− 0.034	− 0.093	^16,54^
Radiation therapy	− 0.060	− 0.055	− 0.133	^16,54^
Chemo with radiation	− 0.090	− 0.149	− 0.336	^16,17^
Multipe recurrent/metastatic disease	− 0.343	− 0.273	− 0.414	^16,54^

*NED* no evidence of disease, *EOL* end of life

### Cost-Effectiveness Analysis

To determine the cost-effectiveness of [^99m^Tc]tilmanocept relative to [^99m^Tc]sulfur colloid, we used the Markov model to estimate the incremental cost-effectiveness ratio (ICER), which was computed as:

$$\mathrm{ICER }=\frac{{C}_{\mathrm{til}}- {C}_{\mathrm{sul}}}{{Q}_{\mathrm{til}}- {Q}_{\mathrm{sul}}}$$

where *C*_til_ is the expected cost of the [^99m^Tc]tilmanocept strategy, *C*_sul_ is the expected cost of the [^99m^Tc]sulfur colloid strategy, *Q*_til_ is the expected QALYs of the [^99m^Tc]tilmanocept strategy, and *Q*_sul_ is the expected QALYs of the [^99m^Tc]sulfur colloid strategy. We considered $100,000 per additional QALY as the threshold for determining cost-effectiveness.^33^

### Sensitivity Analyses

Multiple one-way and two-way sensitivity analyses were conducted to explore the sensitivity of the base case result to changes in model parameters. Each parameter was explored over a plausible range, looking for how sensitive the cost-effectiveness of the base case result was and whether there was a threshold beyond which the optimal decision changed.

## Results

### Base Case Analysis

The base case cost-effectiveness analysis suggested that the [^99m^Tc]tilmanocept strategy was more effective than [^99m^Tc]sulfur colloid by 0.12 QALYs (7.06 versus 6.94 QALYs). [^99m^Tc]Tilmanocept was also more costly, with a lifetime cost of $84,961 in comparison with $84,264 for [^99m^Tc]sulfur colloid. Overall, the base case ICER was $5859 per additional QALY, which was well under the willingness-to-pay (WTP) threshold of 100,000/QALY, and therefore, [^99m^Tc]tilmanocept was deemed cost-effective.

### Sensitivity Analyses

**Fig. 2:**
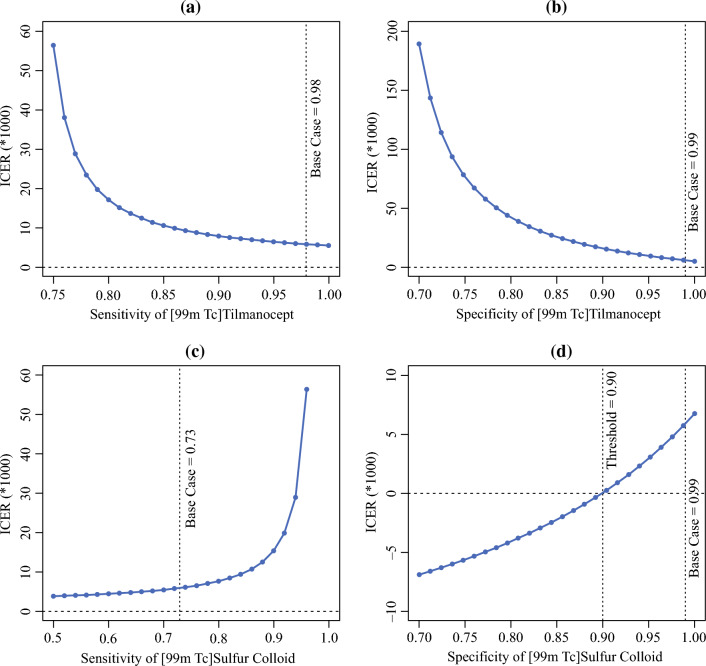
One-way sensitivity analysis of diagnostic oral cavity squamous cell carcinoma (OCSCC), sensitivity and specificity of agents explored over plausible range to assess sensitivity of base case results; *ICER* incremental cost-effectiveness ratio

One-way sensitivity analyses were performed to assess the sensitivity of the models to diagnostic performance characteristics of the agents. The ICER was sensitive to changes in several model parameters (Fig. 2). The [^99m^Tc]tilmanocept strategy maintained its cost-effectiveness despite varying sensitivity of [^99m^Tc]tilmanocept from 0.75 to 1 (Panel a). The ICER decreased with a higher specificity of [^99m^Tc]tilmanocept, and [^99m^Tc]tilmanocept was cost-effective as long as specificity of [^99m^Tc]tilmanocept was higher than 0.73 (Panel b). [^99m^Tc]Tilmanocept remained cost-effective despite widely varying sensitivity of [^99m^Tc]sulfur colloid from 0.5 to 1 (Panel c). [^99m^Tc]Tilmanocept was both less costly and more effective until the specificity of [^99m^Tc]sulfur colloid exceeded 0.90 (Panel d). Above this threshold, [^99m^Tc]tilmanocept still remained cost-effective to varying specificity of [^99m^Tc]sulfur colloid from 0.90 to 1.

**Fig. 3:**
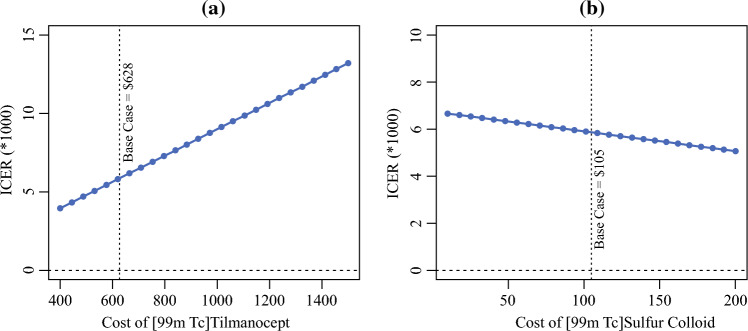
One-way sensitivity analysis of cost of diagnostic OCSCC, cost of agents explored over plausible range to assess sensitivity of base case results; *ICER* incremental cost-effectiveness ratio

One-way sensitivity analyses of the cost of [^99m^Tc]tilmanocept and [^99m^Tc]sulfur colloid are shown in Fig. 3. The ICER was moderately sensitive to variations in the cost of [^99m^Tc]tilmanocept, from $400 to $1500 (Panel a), yet [^99m^Tc]tilmanocept remained cost-effective. [^99m^Tc]Tilmanocept was also cost-effective despite varying the cost of [^99m^Tc] sulfur colloid from $10 to $200 (Panel b).

**Fig. 4:**
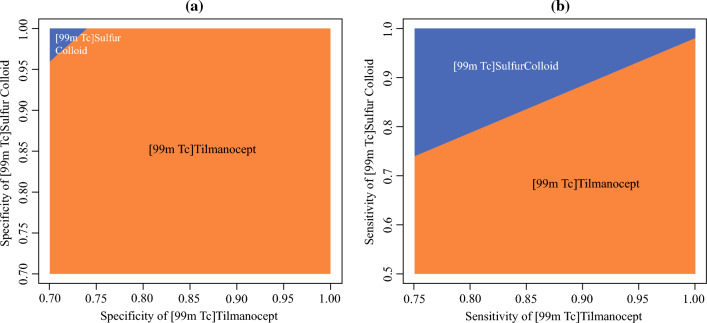
Two-way sensitive analysis of diagnostic OCSCC; two-way analysis of sensitivity, specificity, and cost of agents explored over plausible range to assess sensitivity of base case results

We also conducted two-way sensitivity analyses for sensitivity and specificity of [^99m^Tc]tilmanocept versus [^99m^Tc]sulfur colloid (Fig. 4). [^99m^Tc]Tilmanocept was cost-effective for all values of specificity in the examined range, except when the specificity of [^99m^Tc]sulfur colloid was higher than 0.96 (Panel a). The cost-effectiveness was sensitive to changes in sensitivity of [^99m^Tc]tilmanocept and [^99m^Tc]sulfur colloid (Panel b). [^99m^Tc]Tilmanocept maintained its cost-effectiveness as long as sensitivity of [^99m^Tc]sulfur colloid was less than 0.74. All else equal, whichever agent had a higher sensitivity value was more likely to be cost-effective. If the sensitivity of [^99m^Tc]sulfur colloid was higher than 0.98, [^99m^Tc]sulfur colloid would be cost-effective no matter the sensitivity of [^99m^Tc]tilmanocept. However, 0.98 is far above base values and not consistent with prior literature.^18–24^

## Discussion

The introduction of SLNB for staging of the node-negative neck in early stage OCSCC has allowed surgeons to tailor surgical management and potentially minimize morbidity of surgery for patients who do not require a neck dissection, while targeting those who may be at higher risk for occult nodal metastasis. There have been several multicenter prospective trials that begin to demonstrate the potential therapeutic benefit of SLNB in OCSCC.^38–40^ One multicenter prospective observational study demonstrated the oncologic safety of SLNB in OCSCC, reporting 3-year disease-free survival (DFS) rate of 92%,^38^ while echoing the decreased hospital length of stay and surgical morbidity associated with SLNB shown in other studies.^8,9,41^ Although the prognostic utility of SLN status in the management of early stage OCSCC is actively undergoing further investigation in clinical trials,^42^ the diagnostic benefit of SLNB in early stage OCSCC is clear.^38–40^ However, this benefit relies heavily on the efficacy of the radionuclide agents utilized in this technique. When performing SLNB in the head and neck, the proximity of the primary tumor site to the potential location of the first echelon nodes is technically challenging. This is particularly difficult for floor of mouth tumors, which tend to drain to the nearby level 1a and 1b nodal basins. The smaller size of [^99m^Tc]tilmanocept allows for more rapid clearance from the primary site, while the mannose moiety of [^99m^Tc]tilmanocept allows for targeted binding to the mannose-binding receptor (CD206) expressed on the surface of reticuloendothelial cells within lymph nodes, allowing for improved nodal retention. These characteristics of [^99m^Tc]tilmanocept allow for improved SLNB success in the head and neck with lower false negative rates compared with sulfur colloid. This is particularly relevant, as multiple studies have demonstrated worse overall survival for patients with SLN+ disease, with rates as low as 38%, in comparison with overall survival rates of more than 80% for patients with SLN− disease.^43,44^

Multiple radionuclide agents are used in SLNB, with [^99m^Tc]sulfur colloid and [^99m^Tc]tilmanocept being the most common agents in the USA. Although the estimated proportion of surgeon preference for a particular agent is not published, cost is most likely the primary barrier to the more widespread adoption of [^99m^Tc]tilmanocept in the USA. The most frequently used agent in Europe is [^99m^Tc]nanocolloid, which has a particle size ranging anywhere between 5.6 and 122 nm depending on the manufacturer.^45^ The ideal features of a radiotracer agent allow for rapid clearance from the primary site with persistent retention in the sentinel node. Thus, a key component to the success of an agent in identifying a SLN is the size of the particle: too large of a particle results in decreased transit through the lymphatic channels to the first echelon nodes, resulting in shine-through effect, while too small of a particle could pass through the lymphatic system too quickly. Both options result in increased false negative rates.^45^ One group in Europe performed a within-patient comparison between [^99m^Tc]tilmanocept and [^99m^Tc]nanocolloid, noting a higher injection site clearance for [^99m^Tc]tilmanocept but lower uptake in the SLN.^46^ However, they did note this may be due to a lower injection dose of [^99m^Tc]tilmanocept than [^99m^Tc]nanocolloid (74 MBq versus 120 MBq), and that others have found this was overcome by a higher injection dose of [^99m^Tc]tilmanocept.^46^ Despite the published benefits of [^99m^Tc]tilmanocept in the oral cavity, more recent results have been mixed regarding the superiority of this agent in other disease sites such as the breast. In one comparison between [^99m^Tc]tilmanocept and [^99m^Tc]sulfur colloid for identification of SLNs in breast cancer published in 2015, Baker et al. found the molecular targeting of [^99m^Tc]tilmanocept allowed for fewer SLNs to be removed while still maintaining the same rate of node-positive identification, suggesting the superiority of [^99m^Tc]tilmanocept for breast cancer.^47^ However, it is important to recognize that subsequent studies demonstrated no difference in SLN localization with [^99m^Tc]sulfur colloid when compared with [^99m^Tc]tilmanocept.^48–50^

Several studies have demonstrated other benefits of [^99m^Tc]tilmanocept aside from its physical chemistry properties. [^99m^Tc]sulfur colloid is the most common agent used for SLNB in breast cancer in the USA and injection site pain is a common adverse effect.^51^ As such, many have investigated various ways to decrease injection site pain, such as applying topical lidocaine to the skin or mixing lidocaine with the agent, with mixed results.^52,53^ In a randomized double-blinded single institution controlled clinical trial comparing post-injection site pain of [^99m^Tc]sulfur colloid compared with [^99m^Tc]tilmanocept, patients receiving the [^99m^Tc]sulfur colloid injection reported significantly more pain than those injected with [^99m^Tc]tilmanocept.^51^

To date, there has never been a direct head-to-head comparison of overall outcomes or cost analysis between the two agents. While [^99m^Tc]tilmanocept is more costly upfront, published data, when used for early stage OCSCC, suggest outcomes may be favorable for [^99m^Tc]tilmanocept given the improved false negative rate and negative predictive value compared with [^99m^Tc]sulfur colloid, which would suggest improved outcomes given reduced rates of recurrence and need for additional treatments or surgeries as well as improved quality of life for patients. As a result, one multicenter cooperative group prospective randomized phase II/III clinical trial of SLNB versus elective neck dissection for early stage OCSCC (NRG-HN006; NCT04333537) allows surgeons to choose which agent they prefer to use, and this data will be analyzed at the completion of the trial to further elucidate any potential benefits.^42^ Our analysis of the cost-effectiveness of [^99m^Tc]tilmanocept versus [^99m^Tc]sulfur colloid showed that despite the increased upfront costs of [^99m^Tc]tilmanocept, there was significant QALY gained by its use as demonstrated by the base case ICER of $7933 per additional QALY, which was well under the threshold of $100,000/QALY. As such, these findings support the upfront investment in the higher cost of [^99m^Tc]tilmanocept, given the considerable cost-savings afforded to the healthcare system over a patient’s lifetime. Further studies are warranted to investigate the potential benefits and cost-savings of [^99m^Tc]tilmanocept in other disease sites such as the breast.
